# Predict marital satisfaction based on the variables of socioeconomic status (SES) and social support, mediated by mental health, in women of reproductive age: Path analysis model

**DOI:** 10.1002/brb3.2482

**Published:** 2022-02-09

**Authors:** Farzaneh Rashidi Fakari, Mahbobeh Ahmadi Doulabi, Zohreh Mahmoodi

**Affiliations:** ^1^ Department of Midwifery, School of Medicine North Khorasan University of Medical Sciences Bojnurd Iran; ^2^ Department of Midwifery and Reproductive Health, School of Nursing and Midwifery, Midwifery and Reproductive Health Research Center Shahid Beheshti University of Medical Sciences Tehran Iran; ^3^ Social Determinants of Health Research Center Alborz University of Medical Sciences Karaj Iran

**Keywords:** marital status, mental health, satisfaction, social support, socioeconomic status

## Abstract

**Background:**

The present study aimed to predict marital satisfaction based on the variables of socioeconomic status (SES) and social support, mediated by mental health, in women of reproductive age using the path analysis model.

**Methods:**

This descriptive‐analytical study was conducted on 608 women referred to clinics affiliated to Shahid Beheshti University of Medical Sciences in 2021 using multistage sampling. Data were collected using a personal‐demographic questionnaire, the SES scale developed by Garmaroudi et al. in Iran, the Perceived Social Support scale, the Perceived Stress Scale (PSS), Spielberger State‐Trait Anxiety Inventory (STAI), Beck's Depression Inventory (BDI), and the ENRICH marital satisfaction scale. Data were analyzed in SPSS and LISREL‐1.96 software at a significance level of *p* < .05.

**Results:**

Most surveyed women were aged 21–30 years (50.2%) and were housewives (68%). According to the results of the path analysis test, among the variables that were causally related to marital satisfaction in only one path, depression had the highest negative correlation with marital satisfaction in the direct path (*B* = −0.23), SES the highest positive correlation in the indirect path (*B* = 2.336), and social support the highest positive correlation both in the direct and indirect paths (*B* = 0.365).

**Conclusion:**

The results showed that more favorable social support and SES are associated with higher marital satisfaction while a higher level of depression is associated with a lower marital satisfaction. Therefore, these factors play important roles in marriage sustainability and marital satisfaction.

## Graphical abstract

Marriage and marital satisfaction constitute one of the important areas of human life that require adaptation. Social support and SES are associated with higher marital satisfaction while a higher level of depression is associated with a lower marital satisfaction.

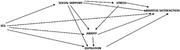

## 1 INTRODUCTION

Marriage and marital satisfaction constitute one of the important areas of human life that require adaptation (Bagarozzi, 2014). Marital satisfaction is a state in which a couple feels happy and satisfied with their marriage and being together (Taniguchi et al., 2006). A satisfying marriage creates a good atmosphere for the intersection and exchange of positive feelings and emotions between the couple. Therefore, the ability to understand and accept each other's thoughts, feelings, and emotions in marital life is associated with a greater sense of satisfaction (Fitness, 2001).

Researchers have conducted many studies to identify the factors affecting marital relationships, conflicts, and problems (Bradbury et al., 2000). The spouse's income and employment status, the couple's high level of education, high socioeconomic status (SES), continuous economic pressures, number of children, and the couple's personality traits are proposed in literature as factors affecting marital relations (Carlson, 2008; Carr, 2012; Vaijayanthimala, Kumari, & Panda, 2004).

Women are more vulnerable in low socioeconomic conditions, and according to studies, women at lower SESs suffer more from depression (Petterson & Albers, 2001). Low‐income women experience greater stress, which can be a predictor of their mental health (Murata et al., 2008). Studies have found a relationship between the family's SES) and perceived stress and the incidence of depression (Rahmani et al., 2011). It has also been reported that the psychological pressure on women decreases as income, job status, and household educational status improve (Seyyedan, 2004).

Another important factor affecting marital satisfaction is social support. This factor affects the maintenance and increase in marital satisfaction by preventing emotional withdrawal, isolation, and depression in stressful stages of life. In addition, in the event of marital conflicts, social support stops conflict from escalating and prevents turning to destructive behaviors. Supportive relationships strengthen the emotional bonds between the couple and lead to a positive marital experience (Cutrona, 1996).

Studies have also shown that psychological factors such as mental health problems, including depression (Grames et al., 2008), stress (Kaleta, 2014), the amount of social support received from the spouse (Acitelli & Antonucci, 1994), and increased anxiety are other predictors of marital dissatisfaction, and the higher the level of depression and anxiety, the lower will be marital satisfaction (Kondajani et al., 2008). Anxiety is one of the harms that affect humans' social and personal life and can act as a stimulus for life activities and be helpful in all conditions; however, it can also be one of the most common symptoms of neuroticism in people and influence humans' life (Raffety et al., 1997).

**FIGURE 1:**
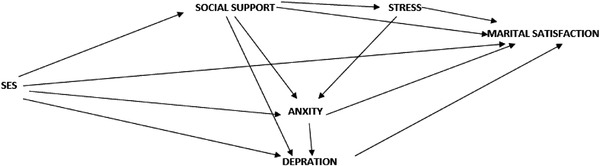
Theoretical model of predicting marital satisfaction based on the variables of socio‐economic status and social support a model mediating women's mental health

According to studies, many internal and external factors certainly affect marital satisfaction and various studies have examined the effects of the variables of SES, social support, and mental health on marital satisfaction; however, all these studies have separately examined the effect of the variables on marital satisfaction. The present study examined these variables interactively in the form of a conceptual model. Consequently, conceptual model No. 1 shows the relationship between the factors affecting marital satisfaction (Figure 1). This study thus seeks to predict marital satisfaction based on SES and social support variables, mediated by mental health, in women of reproductive age using the path analysis model.

## 2 METHODS

### 2.1 Study design and population

This cross‐sectional study was conducted on 608 women of reproductive age who had referred to clinics affiliated to Shahid Beheshti University of Medical Sciences in 2021. The sample size was determined as 608 based on the research variables, 30 observations per independent variable, and 10% sample loss (Plichta et al., 2013).

Multistage sampling was used to select the subjects. First, different regions covered by Shahid Beheshti University of Medical Sciences were designated as the categories. Then, a list of all the covered health clinics in these regions was prepared as the clusters, and some centers were randomly selected from these regions. Then, a quota was allocated to each center based on the population covered.

### 2.2 Inclusion and exclusion criteria

The inclusion criteria were being an Iranian woman aged 18–35 years, having no known acute or chronic diseases based on the self‐reports and the health records, no history of mental disorders in the subject or their family, and not having experienced any major stressful or significant tragic incidents in the past 6 months. The exclusion criterion was failure to complete the questionnaires.

### 2.3 Measuring tools

Data were collected using a personal‐demographic questionnaire, the SES scale developed by Garmaroudi & Moradi (2010). in Iran, the Perceived Social Support scale, the Perceived Stress Scale (PSS), the Spielberger State‐Trait Anxiety Inventory (STAI), Beck's Depression Inventory (BDI), and the ENRICH marital satisfaction scale.

### 2.4 Personal‐demographic questionnaire

The personal‐demographic questionnaire had 20 items. Its validity was measured by the content validity method.

### 2.5 Economic and social status

SES was measured using the questionnaire developed by Garmaroudi and Moradi (2010), which inquired about the mother's and the spouse's education, housing space per person, housing price per square meter, amenities, and computer access. The correlation between these factors and the total score was 0.87 based on this questionnaire. By matching the scores, the cut‐off point of 16 was determined by the summary index to create a two‐state variable and classify households into two groups, including favorable and nonfavorable SES. The maximum potential score in the summary index was 48 (Garmaroudi & Moradi, 2010).

### 2.6 Spielberger state‐trait anxiety inventory

The STAI has separate self‐assessment scales to measure state and trait anxiety. The state anxiety scale consists of 20 statements that assess the subject's feelings “in this moment and time of answering.” The trait anxiety scale has 20 statements that measure the subject's general and ordinary emotions. Based on the answers provided, a weight between 1 and 4 is assigned. A score of 4 indicates high anxiety (Spielberger, 2010). Azimi and Zarghami (2002) reported the reliability of 0.91 and 0.90 for the state and trait anxiety scales, respectively.

### 2.7 Beck depression Inventory‐II

There are 21 items related to domains such as sadness, pessimism, feelings of helplessness and failure, feeling of guilt, sleep disturbance, loss of appetite, and self‐hatred. Two of these items are assigned to emotions, 11 to cognition, two to overt behaviors, five to physical symptoms, and one to interpersonal semiotics (Beck et al., 1988; Wang & Gorenstein, 2013). The internal reliability of this instrument for the Iranian society has been approved with Cronbach's alpha coefficient of .87 and its test–retest reliability has been approved with a score of 0.74 (Ghassemzadeh et al., 2005). Doulabi et al. (2019) reported the reliability of this questionnaire with Cronbach's alpha coefficient of .85 (Doulabi et al., 2019).

### 2.8 Perceived stress scale

The PSS measures thoughts and feelings about stressful events, controlling, overcoming, and coping with stress, and experienced stresses. It also examines the risk factors of behavioral disorders and shows the process of development of stressful relationships (Cohen et al., 1983).

This study used the 14‐item version of PSS. In this tool, the subject must score the items based on a five‐point Likert scale from “never” (= 0) to “very often” (= 4). Its seven negative items indicate the inability to cope with stress and its seven positive items indicate the good adaptation of the subject to stressors. Its lowest score is 0 and its highest score 56, and higher scores on the tool denote higher perceived stress (Cohen et al., 1983). The psychometrics of this questionnaire for the Iranian society were assessed by Maroofzadeh et al. (2014), who reported an internal reliability coefficient of 0.86.

### 2.9 Social support appraisals (SS‐A) scale

This questionnaire has 23 items and measures the size and degree of availability and adequacy of attachment and social cohesion. The questionnaire shows how much a person believes that he is loved and respected by others and the extent of his relationships with his family, friends, and others (Hamid, 2006; Kang et al., 1998). The family subscale has eight items, the friends subscale seven items and the others subscale also has eight items (Vaux et al., 1986). This study used a modified form of the PSS scale. Each “Yes” or “No” option was assigned a value of 1 or 0 depending on the content of the statements. The possible score range for this tool is 0–23 (Hamid, 2006). Rashedi et al. (2013) reported the reliability of the questionnaire with *α* = .83 for overall social support, *α* = .89 for social support from family, *α* = .86 for social support from friends, and *α* = .86 for social support from others (Rashedi et al., 2013).

### 2.10 ENRICH: Marital satisfaction scale (EMS)

The ENRICH marital satisfaction scale is a general measure of marital relationships, including idealistic distortion, marital satisfaction, personality issues, communication, conflict resolution, financial management, leisure activities, sexual relationship, children and parenting, family and friends, equalitarian roles, religious orientation, marital cohesion, and marital change (Asoodeh et al., 2011; Fowers & Olson, 1989). This scale contains 35 items and four subscales, namely marital satisfaction, communication, conflict resolution, and idealistic distortion, which are scored based on a five‐point Likert scale from 1 to 5: “Strongly disagree,” “disagree,” “neither agree nor disagree,” “agree,” and “strongly agree.” Asoodeh reported the alpha coefficient of the questionnaire as 0.86, 0.80, 0.84, and 0.83 for the subscales of marital satisfaction, communication, conflict resolution, and idealistic distortion, respectively, and their test–retest reliability as 0.86, 0.81, 0.90, and 0.92, respectively. Asoodeh (2011) used the scale on 365 couples and reported the alpha coefficient of the questionnaire for the listed subscales as 0.68, 0.78, 0.62, and 0.77, in respective order. The scores of the scales are calculated according to the cut‐off points and the scale is interpreted based on tables of norms and the interpretation guide (Asoodeh et al., 2011).

### 2.11 Data collection

Data collection started after the research proposal was approved and permission was obtained from the ethics committee of Shahid Beheshti University of Medical Sciences (ethics code: IR.SBMU.PHARMACY.REC.1399.329) and other necessary permits were acquired from Shahid Beheshti University of Medical Sciences and the School of Nursing and Midwifery.

First, the researcher attended the selected health centers and explained the study objectives to the women referring to the centers; then, after obtaining their consent, she asked the eligible women to fill in the personal‐demographic questionnaire, the SES scale by Garmaroudi & Moradi, 2010, the Perceived Social Support scale, the PSS, the STAI, BDI, and the ENRICH marital satisfaction scale and to then deliver them to the researcher.

### 2.12 Data analyses

Descriptive statistical tests (frequency distribution, central, and dispersion indices, including mean and standard deviation), and correlation were used for the analysis of the data. LISREL‐8.8 software was used for the path analysis. The significance level was less than 0.05.

## 3 RESULTS

Most women were aged 21–30 years (50.2%), 417 (68%) were housewives, and their husbands were aged 32–41 years (51%) and were company employees (42.6%). The mean score of depression was 9.9 ± 9.6, stress 17.57 ± 3.1, social support 17.57 ± 3.1, and marital satisfaction 112.5 ± 13.74 (Table 1).

**TABLE 1:** Some characteristics studied in socio demographic

		*F* (%)	Mean ± SD
Age women	<20	11(1.8)	30± 5
	21−30	305(50.2)	
	31−40	279(45.9)	
	41−50	13(2.2)	
Age Men	22−31	205(33.7)	35 ± 6.04
	32−41	310(51)	
	42−51	85(14)	
	>52	8(1.4)	
Job Men	Employee	259(42.6)	
	worker	132(21.7)	
	Self ‐ employment	191(31.4)	
	Un employed	26(4.3)	
Job Women	House keeper	417(68)	
Depression	normal	357(58.7)	
	mild	148(24.3)	
	moderate	75(12.3)	
	sever	22(3.6)	
	Very sever	6(1)	
	Mean total ± SD		9.9 ± 9.6
Stress	Mean total ± SD		17.57 ± 3.1
Anxiety	Mean total ± SD		47 ± 6
Social support	Friend support		6.21 ± 1.4
	Family support		6.24 ± 1.3
	Other support		5.1 ± 1.2
	Mean total ± SD		17.57 ± 3.1
Marital Satisfaction			112.5 ± 13.74
Socio‐economic Statues(Score)			20.57 ± 5.5

Based on Pearson's correlation test, social support had the most positive relationship (*r* = .32) and trait anxiety the most negative or inverse relationship (*r* = −.96) with marital satisfaction (Table 2).

**TABLE 2:** Correlation matrix between social support, mental heal, and marital satisfaction

		1	2	3	4	5	6
1	Depression	1					
2	Anxiety	−0.085^*^	1				
3	Social support	−0.255^**^	−0.214^**^	1			
4	SES	−0.173^**^	0.171^**^	−0.095^*^	1		
5	Stress	−0.269^**^	0.201^**^	−0.053	0.098^*^	1	
6	Marital satisfaction	−0.282^**^	−0.96^*^	0.32^*^	0.025	0.005	1

** Correlation is significant at the 0.01 level (2‐tailed).

* Correlation is significant at the 0.05 level (2‐tailed).

**FIGURE 2:**
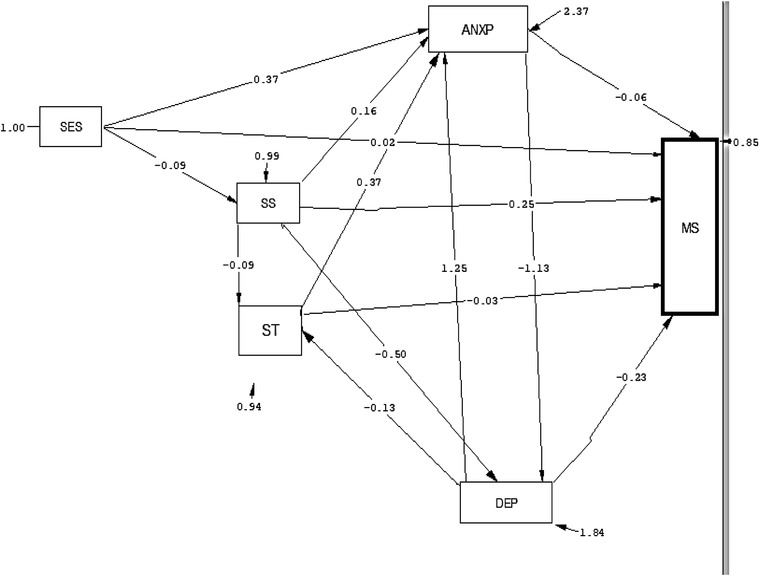
Testing the path analysis model of the social support, mental heal, and marital satisfaction

Based on the path analysis test (Figure 2), among the variables that were causally related to marital satisfaction in only one path, depression had the highest negative correlation with marital satisfaction (*B* = −0.23) in the direct path, SES the highest positive correlation in the indirect path (*B* = 2.336) and social support the highest positive correlation both in the direct and indirect paths (*B* = 0.365). In other words, more favorable social support and SES were associated with a higher marital satisfaction, and a higher level of depression was associated with lower marital satisfaction (Table 3).

**TABLE 3:** Total effect of variable on Marital Satisfaction

	Direct Effect	In Direct Effect	Total Effect
ANXP	−0.06	0.26^∗^	0.26^∗^
SS	0.25^∗^	0.115^∗^	0.365^∗^
ST	0.03	0.1^∗^	0.1^∗^
SES	0.02	2.336^∗^	2.336
DEP	−0.23^∗^	–	−0.23^∗^

^∗^ Statistically significant ANXP, Anxiety; DEP, Depression; SES, Social economic statues; SS, Social Support; ST, Stress.

The model fit indices showed the desirability and high fit of the model and the reasonable relationships between the variables based on the conceptual model (Table 4).

**TABLE 4:** Characteristics of the goodness of fit of path analysis model

Model	*X* ^2^	Df	CFI	GFI	NFI	RMSEA	AGFI
	3.63	1	0.99	1	0.99	0.56	0.96

*Abbreviations*: ; AGFI, adjusted goodness of fit index; CFI, comparative Fit Index; df, degree of freedom; GFI, goodness of fit index; RMSEA, root mean square error of approximation.

## 4 DISCUSSION

This study was conducted to predict marital satisfaction based on the variables of SES and social support mediated by mental health in women of reproductive age.

The results suggested that more favorable social support and SES were associated with a higher marital satisfaction.

Lampis et al. (2021) showed that people with lower levels of satisfaction and support suffer more psychological, interpersonal, and social problems. Panahi et al. (2018) showed that social support has a direct positive effect on marital satisfaction. Social support has a buffering effect in unpleasant situations and psychological damage and acts by influencing the intervening variables, such as coping with the effects of negative situations or stressful events (Roohafza et al., 2016). Therefore, one can expect that as the power of this psychological buffer increases, marital relations also become stronger and marital satisfaction also increases in parallel to it. In general, it can be argued that enjoying social support makes it easier to solve problems and people with higher social support can more easily solve their issues. Social support also increases the couple's self‐confidence and is effective in dealing with life stresses (Rostami et al., 2013). Furthermore, receiving emotional support from others increases the quality of life and promotes mental health (Gamari et al., 2014).

Lichter and Carmalt (2009) showed that poor economic status and low income have adverse effects on the quality and stability of marriage. Dobrowolska et al. (2020) showed a relationship between economic status and marital satisfaction in different cultures. In a poor economic status, when the basic needs are not met, couples may pay less attention to intimacy (Jackson et al., 2016). Therefore, a poor economic status may be negatively correlated with marital satisfaction (Conger et al., 1990).

Appropriate financial and economic resources are environmental factors that, according to many scientists, dominate all aspects of human life. A favorable economic or financial situation has always been considered a very significant factor affecting individuals’ and families’ health. The economy can be effective not only in choosing a spouse, but also in maintaining the marital relationship and increasing its quality (Kinnunen & Feldt, 2004). An unfavorable financial status has a devastating effect on the marital relationship, to the extent that having a low financial status in comparison with one's relatives and neighbors can deter one's relationship (Cutrona et al., 2003).

The results of the present study suggest that higher depression levels are associated with lower marital satisfaction. Papp et al. (2007) showed that spouses’ psychological disturbances, such as depression and anxiety, are simultaneously associated with conflicts and increased negative emotions, such as anger, sadness, and grief. In addition, depressed people are more likely to withdraw from each other during marital conflicts, and this reduced likelihood of conflict resolution may itself contribute to their depressive symptoms (Papp et al., 2007).

Kouros and Cummings showed that the husband's depression is a predictor of increased depression in the wife over time. This relationship was stronger in couples who reported marital turmoil than in couples who reported higher marital satisfaction (Kouros et al., 2010). Negative marital experiences of this type become a source of stress and depression for couples. In these negative and stressful situations, the couple's manifestation of depression symptoms is not improbable. Studies have also shown that couples with depressed spouses themselves express a depressed mood, negative verbal and nonverbal behavior, and psychological and physical complaints in their marital interactions (Papp et al., 2009).

Among the limitations of the present study was that some participants may not have given completely correct answers to some of the items in the questionnaires. Nonetheless, we tried to ameliorate this issue in our data collection by assuring the participants about the confidentiality of their responses and the importance of their honest cooperation in answering the questionnaires.

## 5 CONCLUSION

The present findings showed that more favorable social support and SES were associated with a higher marital satisfaction while a higher level of depression was associated with lower marital satisfaction. Therefore, these factors play an important role in marriage sustainability and marital satisfaction.

## CONFLICT OF INTEREST

None.

### PEER REVIEW

The peer review history for this article is available at https://publons.com/publon/10.1002/brb3.2482
